# Reciprocity between States

**Published:** 1908-11

**Authors:** A. A. Kent

**Affiliations:** Lenoir, N. C., President of the Board of Medical Examiners of the State of North Carolina, and Delegate for North Carolina to the American Confederation of Reciprocation and Examining Licensing Medical Boards


### RECIPROCITY BETWEEN STATES.

BY A. A. KENT, M. D., LENOIR, N. C., PRESIDENT OF THE BOARD OF MEDICAL EXAMINERS OF THE STATE OF NORTH CAROLINA, AND DELEGATE FOR NORTH CAROLINA TO THE AMERICAN CONFEDERATION OF RECIPROCATION AND EXAMINING LICENSING MEDICAL BOARDS.

Reciprocity between states as applied to the practice of medicine means an agreement between two state boards of examiners by which each will recognize the licns granted by the other and grant to the holder of such license from one state the license to practice medicine in the other state without a second examination. For example, the Iowa board will recognize the license granted by the North Carolina board and grant a license to practice in the State of Iowa to an applicant holding a license from the North Carolina board without requiring the applicant to stand an examination before the Iowa board. This, upon the surface seems to be a very simple proposition, and much to be desired by doctors moving from one state to another, as only a very few of the best qualified doctors could pass these examination after they have been out in practice a few years, without first taking a special course of study to prepare themselves again upon the fundamental branches At first we wonder why it has not long ago become common custom. When we investigate the question, we find it to be attended with many obstacles, complications and dangers, some of which are almost insurmountable.

If there were but a few states and all had for years maintained a somewhat uniform standard of requirement, and if all applicants were capable and honest, it would be an easy proposition. But we must remember that there are now in the United States 51 political divisions, and that the conditions and environments in these, many divisions differ widely, making very different standards of requirement necessary. The legislation in each has grown up somewhat spordically, many times more in conformity to the ideas of the laity and non-professional legislators, than to the ideas of medical men. Some states have a single board, in some there are mixed boards, in others two or three boards, our state having two boards at this time. In some the different sects are recognized in one way, in others in some other way. In some a high standard has been rigidly maintained for a long time, in others for only a short while, and in yet others such a standard has not been attained. While the legislation of the several states has a general tendency upward and towrd uniformity, it is more or less chaotic and unstabile. Such being the general condition throughout the states, resiprocity should be embraced by North Carolina with a great deal of careful reserve and prudence.

Every state has absolute control of its own domain in all matters of police regulation, the practice of medicine being one of them. It has the right to fix the standard that it will require of its own citizens and other primarily licensed in the state. It also has the right to say whether or not it will recognize the license granted in another state or not, and if so, to fix the terms upon which it will grant such recognition. This power has generally been vested by the states in a board of health or a board of mediical examiners.

The Board of Medical Examiners of North Carolina, having been created by act of the legislature of 1858-1859, is the oldest medical examining board that has been in continuous existence in any of the states. The medical profession of the state has always enjoyed great privileges and likewise great responsibilities. We control ourselves, and have been the authors of all important laws governing and promoting the practice of medicine in the state. The doctors have likewise been the authors of all important legislation of the state for the protection of the public health While we have incidentally safeguarded the interests of the profession, we have always made the protection of the people and the public health of the state of first importance. The conditions and environments of the state being considered, our medical laws are about as good as we could wish for. We have also reached the time when our state boys, who are homogenious with the people, are not only supplying the needs of the state, but are crowding one another in the profesison. We do not need to open a new and easy way of entrance into the profession in order to supplement the supply of doctors.

But, we are living in an age of progress, and must keep pace with the times. While we must safeguard with jealous care against the entrance of undesirable outsiders, we must not shut ourselves within a wall, refusing to recognize the better class of doctors who come to us from other states, thus creating the impression that we are trying to maintain a monopoly, and working prejudice against our own licentiates, who will in the future be moved to other states in greater numbers than we receive in return.

This matter of reciprocity is with us and will not be brushed aside or smothered down, even if we desire to do so. In many of the states it is an established fact, and in common practice. As many as 50 reciprocal license per year have been granted by some of the states. It is in keeping with the greatly increased facilities for better and more general knowledge n regard to all sections of the country. Sentiment in favor of it is growing rapidly in all the states. There are at this time some five or six national organizations that come together annually and discuss it with a view to establishing a uniform standard of requirement and general reciprocity. There exists such a diversity of environment that it will be many years before such a uniform standard can be secured and maintained in all the states. But the work along this line has disseminated much knowledge, and has created such a wide-spread and popular demand upon us at this time as can scarcely be resisted.

The law which was enacted by our last legislature upon this subject was in obedience to this popular demand, and not desired by the profession in the state. In fact it was resisted by the profession upon the belief that the demand at that time was being made by a few undesirable persons, who hoped by means of reciprocity to evade our careful examinations and obtain an easy entrance to the profession in the state. In fact, there was no evidence at that time that there was a general desire on part of the people to open new and easy portals into the profession, and thus meet an demand for an increase in the supply of doctors for the state. However that may have been, we were in great danger of unconditional recognition of all license of the other states, without discrimination, being forced upon us by act of the legislature. Thanks and praise be to Dr. R. H. Lewis, of Raleigh, that this legislation was so directed as to leave the matter of reciprocity in the discretion of the State Board of Medical Examiners. We were very fortunate in having a wise and patriotic member of the profession in our capital city at that time, as in many other instances, to take care of our interests. If the effort to force unconditional recognition of the licenses of all the other states upon us had been successful, North Carolina would soon have been flooded with the undesirable from all sections of the country.

Your Board of Examiners at its next meeting formulated a set of rules for reciprocity that would admit to the profession in this state those applicants from other states holding a license from states having a standard of requirement equal to our own. To have admitted those who had obtained a license for a state with a lower standard would obviously be discriminating against those who were offering to pass before our own board, against our own boys. This would be unjust, and would ultimately force our boys to go before the boards in other states with lower standards, and then come to North Carolina and demand that the license from the other states shall be recognized As our standard is higher than that of all but two or three states we could only reciprocate in a very limited way. We can with safety say we will recognize the license of an applicant from another state, who upon examination made a grade as high or higher than our standard of requirement. This would enable us to reciprocate with most of the states; and would be greatly to the advantage of our licentiates who wish to remove to states having a lower standard than ours.

The matter of reciprocity or recognition of the license granted by another state board should always be put in the hands of the state licensing board. While there should be some conference and general agreement on part of our state board with the boards of other states as to reciprocity, it should be understood by both that it is to be reciprocity for the benefit of the *bona fide* doctors of each state of the desirable class, and not for the purpose of opening an easy avenue into the profession in either state of the undesirable class. We only want the better class who may come to us from other states, and not just any who may hold a license. On the other hand, we should be discreet in the class of men that we recommend to the other states for reciprocity. There are good and bad in the medical profession as well as in other avocations in all of the states. We should stand ready to receive the good, and at the same time discriminate against the incapable and the bad. It should always be kept in mind that the undesirable are apt to be the first to try to avail themselves of it. They will always be the most numerous and the most clamorous for it.

Our board should have a very complete and comprehensive form for the applicant to fill out upon making his application. Without this very important matters of requirement are apt to be lost sight of. No license obtained other than by examination should ever be recognized by our state. The photograph system for indentification should be adopted and strictly adhered to; and all other safeguards against cheating and fraud employed. It would be well for the secretary of the board to be empowered to administer an oath to all applicants for examination as well as reciprocity to the effect that he is the person that he represents himself to be, that he is the rightful owner of the license and diploma which he holds and that he is not adicted to any drink or drug habit to the extent of impairing his usefulness as a practicing physician.

No state board should ever undertake to examine the recently graduated citizens of another state and recommend them for reciprocity; nor should any state ever allow its citizens who have recently graduated to obtain license before the board in a sister state and then come to the home state and demand recognition on the grounds of recipocity. If one could go before the board in a sister state, pass the examinations and obtain a license that would entitle him to practice in the home state, there would soon be a scramble to get before the board that had a reputation for being easy to pass. There would be no chance for the preferment of charges against the man of bad character, for no one could know when or what board he would appear before. Substituting and other cheating could be carried on with less risk of detection. It would always be restricted to individual cases, and exercised upon a high plane. If properly conducted upon a high level of professional honor, dignity and courtesy it will be of great advantage to the worthy doctor who is moving from one state to another. It should be well understood that it is for his benefit, and for him only. It will not only save him great embarrassment, but also the expense, labor and time necessary to preparation for a second examination.

